# Bimodal Solutions or Twice-Daily Icodextrin to Enhance Ultrafiltration in Peritoneal Dialysis Patients

**DOI:** 10.1155/2013/424915

**Published:** 2013-01-08

**Authors:** Periklis Dousdampanis, Konstantina Trigka, Joanne M. Bargman

**Affiliations:** ^1^Hemodialysis Unit, Kyanous Stavros Patras, Germanou 115, 26225 Patras, Greece; ^2^Peritoneal Dialysis Program, University Health Network, University of Toronto, ON, Canada

## Abstract

The efficacy and safety of icodextrin has been well established. In this paper, we will discuss the pharmacokinetics and biocompatibility of icodextrin and its clinical effect on fluid management in peritoneal dialysis patients. Novel strategies for its prescription for peritoneal dialysis patients with inadequate ultrafiltration are reviewed.

## 1. Introduction 

The use of icodextrin (ico) has been characterized as one of the major achievements in peritoneal dialysis (PD) [1]. Ico-based peritoneal dialysis solutions have been used successfully by PD practitioners for two decades.

 Ico is an isoosmolar alternative osmotic agent that induces ultrafiltration (UF) in peritoneal dialysis by colloid osmosis. Peritoneal absorption of ico is limited and occurs by convection *via* the lymphatics of the peritoneum [2]. As a result, the net pressure gradient is relatively constant, sustaining UF for the long dwell. Many clinical benefits of ico have been described, such as a reduction in total glucose load [3], equivalent or higher UF than that provided by hypertonic glucose solutions [4], and better control of fluid balance [5]. Ico is recommended for patients with poor UF and those with a high or high/high-average pattern in the peritoneal equilibrium test (PET). It is well known that UF volume correlates with patient and technique survival [6]. 

Glucose degradation products (GDPs) and the products of advanced glycosylation end products (AGEs) induce inflammation and fibrosis of the peritoneal membrane [7]. Minimizing dextrose exposure by using ico for the long dwell may prevent long-term detrimental changes of the peritoneal membrane.

 In addition, there is a growing concern about the total amount of absorbed glucose and so there is interest in the use of new alternative glucose-sparing osmotic agents.

The use of a “bimodal” solution composed of glucose and ico, in order to increase sodium and fluid removal, is a promising approach [8].

 Ico was used initially during the long night dwell in continuous ambulatory peritoneal dialysis (CAPD) and during the day dwell in continuous cyclic peritoneal dialysis (CCPD). Recently, the daily use of two ico exchanges has been suggested in order to minimize the glucose load and/or to increase the UF rate [9–12]. 

The biocompatibility of ico has been investigated; however, it should be noted that there are data suggesting that those who use icodextrin are still vulnerable to develop encapsulating peritoneal sclerosis (EPS) [13]. 

## 2. Pharmacokinetics of Icodextrin

Ico consists of a complex mixture of starch-derived water-soluble glucose polymers, with varying chain lengths [14, 15]. 

Moberly et al. have observed that a median of 40% of the total administered dose of ico (2 L of 7.5%) was absorbed by lymphatics of peritoneal cavity during the 12 hours [15]. Thus the absorption of ico is slower than that of glucose, rendering this osmotic agent unique due to the longer duration of the net pressure gradient.

Moreover, the plasma levels of ico and its metabolites increase during the dwell and decrease after drain as a result of absorption by the peritoneal lymphatics and their elimination by dialysis [15]. More than 20% of the absorbed icodextrin and metabolites are eliminated by renal excretion and dialysis [15].

The circulating enzyme *α*-amylase hydrolyzes the absorbed ico and its metabolites such as maltose, maltotriose, and maltotetraose, which can subsequently be metabolized to glucose by tissue maltases, or eliminated by urine and dialysis [16]. The intracellular metabolism of maltose and other similar metabolites into glucose does not result in hyperglycemia, because the major amount of glucose produced remains inside the cell.

Data from clinical studies in adults and children on PD that used 7.5% ico-based solutions for the long dwell demonstrate that the concentration of the metabolites increases initially, reaching a steady-state level after 7–10 days [17–21].

Rodríguez-Carmona et al. have investigated the total concentration of circulating ico metabolites in 12 PD patients, in which two ico exchanges were used during nocturnal automated peritoneal dialysis. The authors reported that the total concentrations of ico metabolites did not differ significantly compared to use of one daily ico exchange [22]. 

More recently, Gobin et al. used two ico exchanges daily and found a doubling of the total ico concentration in plasma within 3 months of treatment (345 ± 145 mg/dL to 615 ± 120 mg/dL), which remained stable after six months [9].

Moreover, Sav et al. observed that after using 3 for months two ico exchanges per day, there was a slight increase of blood levels of icodextrin and maltose, but was not statistically significant [10].

In a more recent retrospective study, it has been reported that six months after administration of twice-daily ico exchanges in 8 PD patients, the levels of icodextrin metabolites did not increase significantly [12]. The differences among these studies regarding the concentration of icodextrin and their metabolites could be explained by the limited number of patients enrolled in these studies, by the different duration of each study and by the total number of patients with residual renal function and their amount of urine output (which would serve to excrete the metabolites). 

Posthuma et al. were the first to report that the ico metabolites may increase the serum osmolality [23]. Additionally, Ota et al. confirmed these findings and they reported that the increase of serum osmolality by the circulating ico metabolites had no effect on net UF [20]. 

## 3. Biocompatibility of Icodextrin

Four principal parameters of a PD solution may affect the degree of biocompatibility. These include the pH/buffer system, the osmolality, the concentration of glucose, and the glucose degradation products (GDPs).

All these parameters of a PD solution may activate the cells of the local immune system of the peritoneum. The activation of these cells leads to an increase of proinflammatory cytokines and chemokines resulting in an activation of the proinflammatory and profibrotic pathways [24]. 

 It is well known that uremia, in combination with long-term exposure to bioincompatible solutions, induces structural peritoneal membrane changes, such as denudation of mesothelium, submesothelial thickening, neovascularization, and vascular hyalinization [25]. All these deleterious changes may be mediated by the presence of glucose in conventional PD solutions. GDPs may exert local toxicity leading to mesothelial cell injury [26]. In addition, AGEs increase the vascular permeability and may lead to peritoneal membrane injury [27]. 

Thus, there is an increasing awareness of glucotoxicity with an emphasis on the development of new more biocompatible PD solutions. 

Ico-based solutions which use lactate as a buffer are isoosmolar (282 mOsm), contain low levels of GDPs, and are considered to be more biocompatible than glucose-based conventional solutions, which contain GDPs. The only parameter which is not corrected is the low pH (it is relatively acidic pH = 5.8).

There *ex vivo* and *in vitro* studies that suggest better biocompatibility for ico-based solutions compared to conventional PD solutions [24, 28, 29].

Bajo et al. have shown that ico effluent induces a greater proliferation of human mesothelial cells in comparison with glucose effluent [30]. This is assumed to be a beneficial effect, but is not proven.

However, there are conflicting data regarding ico and peritoneal inflammation [31–33]. In addition, there have been several reports that ico induces allergic responses such as exfoliative dermatitis and sterile peritonitis [34, 35]. 

 There are data from large retrospective studies that implicate icodextrin in the development of EPS [13, 36, 37]. Indeed, although use of icodextrin can reduce the total amount of glucose exposure to the peritoneal membrane, in return the membrane is being exposed to a starch-derived solution that may not necessarily be any more protective of long-term membrane function.

The interpretation of these findings is difficult because the majority of patients had been using glucose-based solutions and ico in tandem. In addition, hypertonic glucose-based solutions were replaced by ico but there was a continuous exposure to glucose due to the use of glucose-based solution with lower tonicity. 

 The role of ico in the extension of technique survival, and the increase of UF rate in PD patients with UF failure is well established. Insofar as the use of ico can keep the patient on PD for a longer time, the association of ico with membrane failure may be the result of the longer duration of PD and nothing about the solution itself [38]. It is worth noting that the biocompatibility of bimodal solutions or the double dose of ico has not been yet investigated. 

## 4. Effect of Icodextrin on Volume Status, Sodium Balance, and Blood Pressure

Volume overload is the major risk factor for hypertension and cardiovascular disease. Fluid overload, due to inadequate UF and/or inadequate control of sodium balance, leads to hypertension, left ventricular hypertrophy, and associated increased mortality. The efficacy of ico on UF and fluid management has been demonstrated [17].

 Finkelstein et al. reported that in patients on APD with high transport characteristics, ico is superior compared to hypertonic solutions for net UF [4].

 Data from two randomized controlled studies reported a reduction in extracellular water (ECW) and total body water (TBW) with the use of ico [5, 19].

 In addition, Konings et al. reported in a group of patients who used ico a decrease in left ventricular mass but not in the blood pressure [5]. Woodrow et al. observed a reduction in ECW and TBW with concomitant reduction in the systolic blood pressure in APD patients which switched from glucose-based solutions to ico [39].

Plum et al. reported that the use of ico increases the sodium removal in patients on APD [19]. The increased peritoneal sodium removal probably reflects both the enhanced UF obtained by colloid osmosis and a decreased sodium sieving. Ultrafiltration by crystalloid osmosis, as done with conventional PD solutions, removes water through both the small interendothelial pores and through aquaporins. Approximately half the water ultrafiltered is done so through the aquaporins. In contrast, the ultrafiltration with ico is through the small pores only, so that the ultrafiltrate is comprised of both water and sodium. Additionally, Fourtounas et al. reported that the use of ico enhances sodium removal in both continuous CAPD and CCPD [40].

 More recently, data from a 12-month, multicenter, open-label controlled trial, reported that ico improves the metabolic control and the fluid management increasing the net UF in high and high-average diabetic patients on CAPD. The authors reported a statistically significant decrease of systolic and diastolic blood pressure in the group using ico [41].

 There are controversial reports regarding the impact of ico on residual renal function [22, 42, 43]. A decline in urine volume which was reported in some studies reflecting the extracellular fluid volume depletion due to the increased UF obtained by ico. However, if ico is used to restore euvolemia in the fluid-overloaded patient, and not to induce volume depletion, residual kidney function is unchanged [44].

## 5. New Therapeutic Strategies to Prescribing Icodextrin

### 5.1. Combined Solutions with Icodextrin and Glucose

Peers was the first to introduce the concept of a mixed solution of ico and glucose [45].Theoretically, the advantages of this combination are sparing glucose and increasing UF, due to the combined effect of crystalloid and colloid osmosis during the same exchange. 

Jenkins and Wilkie compared the UF profile of 1.36% glucose, 3.86% glucose, 7.5% ico and the combination solution of 1.36% glucose plus 7.5% ico in seven patients on CAPD and four patients on APD in a prospective open study [42]. The authors reported an improved UF profile for the combination solution, with similar UF compared to that obtained with the hypertonic 3.86% glucose-based solution. In addition, the combination of the two agents was well tolerated [42].

 Dallas et al. in a 4-week, prospective randomized crossover study with five patients on CAPD and 3 patients on APD, reported that the use of the combination dialysate (7.5% ico plus 1.36% glucose) for the long dwell resulted in a 33% increase in long-dwell UF and a 29% increase in total drain volume in comparison with 7.5% ico alone [46]. Freida et al. have investigated the effect on net UF and sodium removal during a 15-hour single-dwell exchange using, alternatively, 3.86% glucose, 7.5% ico and a combination solution with 2.61% glucose, 6.8% ico with low sodium concentration (121 mmol/L) in seven stable patients on APD [47]. The authors reported that the major advantage of this approach was the enhanced UF (mean 990 mL) and the sodium removal (mean 158 mmol) during the 15 hour-long dwell exchange, obtained by the combination fluid [47]. 

The same investigators studied sodium and water removal obtained by the combined 6.8% ico, and 2.6% glucose solution in twenty-one patients on APD with high transport profile, in comparison with sodium and water removal obtained by 7.5% ico alone. All the patients enrolled in the study were on once-daily ico dwell for at least one month at baseline [8]. The authors reported that the estimated mean percent change from baseline in net UF for the combined solution was 150%, versus 18% for ico based solution (*P* < 0.001), and that the estimated mean percent change from baseline in sodium removal for the combined solution was 147% versus 23% for ico (*P* < 0.001) [8]. It should be noted that the reason for the change of estimated mean percent in net UF and in sodium removal with ico remain obscure in this study. However, the increase in sodium and fluid removal observed in the group using the combined solution was probably the result, in large part, of the low concentration in sodium of the dialysis fluid (121 mEq/L) resulting in greater diffusive flux of sodium into the dialysis solution. 

In support of this hypothesis, Davies et al. reported a favorable effect of low-sodium PD solutions on sodium and water removal resulting in a decline in extracellular water [48]. 

 Galach et al. reported that computer simulations in accordance with clinical data have shown an increase in UF and sodium removal in the group of combined solution with low sodium concentration in comparison with hypertonic solutions and ico-based solutions both with standard sodium concentrations [49]. In contrast, Akonour et al. used the same mathematical model and could not confirm the findings of Galach [49, 50]. 

However, the use of bimodal solutions with low sodium alternative concentration in PD patients with UF failure may represent an alternative option in order to enhance UF. A potential risk of this approach could be considered a concomitant hyponatremia due to increased sodium removal. 

## 6. Twice-Daily Icodextrin Exchanges

For a long time, only one daily exchange with ico during the long dwell has been recommended, in order to increase UF in PD patients with inadequate UF. 

The use of ico during the long dwell was based on the observation (derived from the use of computer simulation) that icodextrin did not produce sufficient UF during the early part of the dwell, whereas it leads to slow but steadily increasing net ultrafiltration after 8 hours. 

Moreover, according to Rippe and Levin, UF obtained by ico continues linear and slightly even after a 15-hour dwell [51]. 

In contrast, Jeloka et al. reported that ico UF does not increase past a 10-hour dwell in APD patients. The authors reported that UF obtained by ico at 8 and 10 hours dwell time are similar and did not change significantly at 14 hours [52].

Since UF does not increase substantially with ico after 8–10 hours, the hypothesis in which this new approach was based is that the two 8-hour exchanges will provide more UF than one exchange over 16 hours.

Gobin et al. were the first to used two ico exchanges per day, in order to reduce glucose exposure in a group of nine patients on APD with high transport characteristics [9]. The authors observed at 6 months of treatment a significant decrease in the patient's glucose exposure from 410 ± 75 to 300 ± 75 g/day [9]. The authors did not observe an increase in UF due to the scheduled dwell of the two ico exchanges (one ico exchange for 4-5 hours and the other for 9-10 hours dwell time).

A recent prospective randomized study in 40 patients on CAPD with UF failure investigated the effect of two ico exchanges on body weight and left ventricular mass index [10]. Twenty patients were on one ico exchange for a 16 h dwell time and twenty patients were on two ico exchanges for 8 h each other dwell time. All the patients enrolled in the study were exposed to ico for the first time.

The authors reported in the group of two ico per day, a decrease in the body weight at the third month of treatment (68 ± 12.30 at baseline to 66.10 ± 11.90 at third month, *P* < 0.05). Moreover, significantly decreased left ventricular mass index was observed in the group with the two ico exchanges [10].

Recently, The PD Unit of the Toronto General Hospital reported its experience in nine PD patients with ultrafiltration failure and weight gain in whom two ico exchanges per day were prescribed [11]. Five CAPD patients received twice daily 7.5% ico-based solution, each with an 8 h dwell time. Each exchange with ico was alternated with dextrose based solution with 4 h dwell time. Four patients were on CCPD using dextrose-based solutions during the night for 8 h and two 7.5% ico-based solutions during the daytime. After six months of therapy, the authors observed a decrease in body weight in six patients by an average of 2.9 ± 1.2 kg. In addition, a decrease in the mean blood pressure was observed in all patients. The new regimen was well tolerated and none of the patients reported any side effects [11].

In another recent study, 28 patients with high transport profile and UF failure on CAPD were randomized to receive either one or two ico exchanges per day. Both groups experienced a decrease in serum brain natriuretic peptide, left ventricular mass, heart rate and cardiothoracic index [53]. Moreover, the authors reported an increase in ejection fraction at eight weeks in both groups. The percentage of change of all the parameters was enhanced in the group of patients who received twice-daily ico exchanges [53].

In a retrospective study, 8 PD patients with inadequate UF were switched from one ico exchange to twice daily ico exchanges. There was a significant increase in net UF from 452 ± 800.5 mL at 1 week before treatment, to 993.9 ± 553.1 mL at 3 months and 1078.1 ± 500.9 mL, observed after 6 months [12]. Moreover, osmolality and residual urinary output remained unchanged throughout the study [12].

There is a growing interest in the use of twice-daily ico exchanges either to reduce glucose load or to enhance UF. Recently, the Canadian Society of Nephrology work group suggested the use of two ico exchanges in PD patients with UF failure in order to enhance UF [54].

However, an important issue that must be addressed is whether the accumulated ico metabolites over the long term have an increased potential risk to peritoneal membrane function and systemic toxicity. More attention and further exploration are needed, given the findings regarding the doubling of the serum concentration of ico in the study of Gobin et al. [9]. At present, there is no reported systematic toxicity due to ico or its metabolites. After almost two decades of use, toxicity has become less of a concern for the PD community. 

Twice-daily ico prescription has not been approved by the pharmaceutical agencies and more studies are needed to prove the safety and the efficacy of this new regimen. In addition, the increased cost of this regimen must be considered, although it should be compared to a transfer to hemodialysis [11].

In our opinion, use of bimodal solutions or twice-daily icodextrin exchanges may be warranted. It is imperative to provide adequate UF, especially in anuric PD patients, in order to maintain euvolemia and likely extend survival. 

## 7. Conclusion

Ico is safe and effective in PD patients. Patients with UF failure may benefit from the use of combined solutions or from the use of two ico dwells daily. Further studies are needed to document the efficacy and safety of these new strategies.
